# Functions and specificity of bacterial carbohydrate sulfatases targeting host glycans

**DOI:** 10.1042/EBC20220120

**Published:** 2023-04-18

**Authors:** Ana S. Luis, Edwin A. Yates, Alan Cartmell

**Affiliations:** 1 Department of Medical Biochemistry and Cell Biology, University of Gothenburg, Box 440, 405 30 Gothenburg, Sweden; 2 Department of Biochemistry and Systems Biology, Institute of Systems, Molecular and Integrative Biology, University of Liverpool, Liverpool L69 3BX, U.K.

**Keywords:** Carbohydrate sulfatases, glycosaminoglycans, Human gut microbiota, Mucin, structural biology

## Abstract

Sulfated host glycans (mucin *O*-glycans and glycosaminoglycans [GAGs]) are critical nutrient sources and colonisation factors for Bacteroidetes of the human gut microbiota (HGM); a complex ecosystem comprising essential microorganisms that coevolved with humans to serve important roles in pathogen protection, immune signalling, and host nutrition. Carbohydrate sulfatases are essential enzymes to access sulfated host glycans and are capable of exquisite regio- and stereo-selective substrate recognition. In these enzymes, the common recognition features of each subfamily are correlated with their genomic and environmental context. The *exo*-acting carbohydrate sulfatases are attractive drug targets amenable to small-molecule screening and subsequent engineering, and their high specificity will help elucidate the role of glycan sulfation in health and disease. Inhibition of carbohydrate sulfatases provides potential routes to control Bacteroidetes growth and to explore the influence of host glycan metabolism by Bacteroidetes on the HGM ecosystem. The roles of carbohydrate sulfatases from the HGM organism *Bacteroides thetaiotaomicron* and the soil isolated *Pedobacter heparinus* (*P. heparinus*) in sulfated host glycan metabolism are examined and contrasted, and the structural features underpinning glycan recognition and specificity explored.

## Introduction

**Figure 1:**
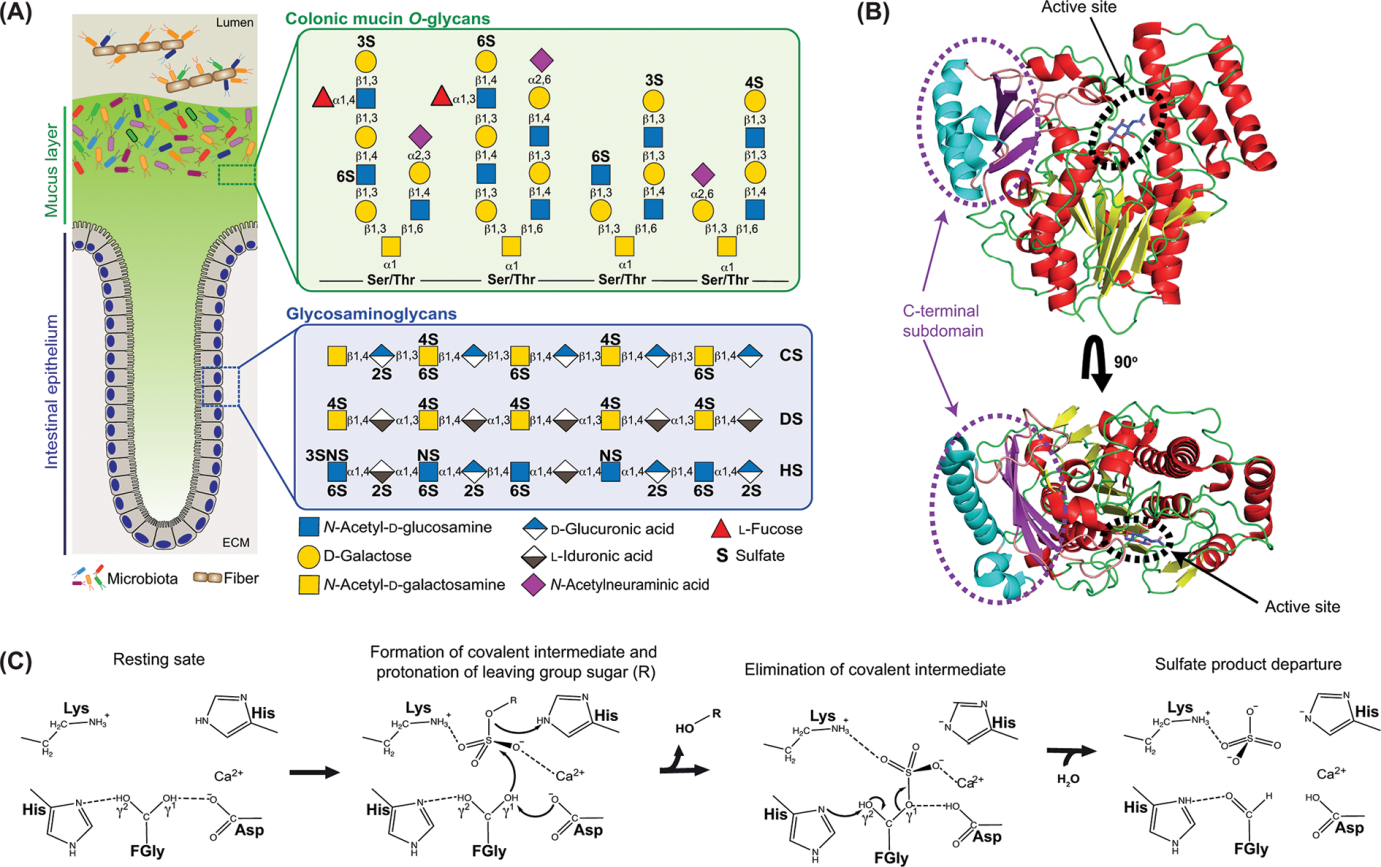
Structure of human-sulfated glycans and S1 sulfatase structure and mechanism (**A**) The structure of cMOs (green background) and the structure of the common GAGs (peach background). (**B**) The N-terminal α-/-β-/-α alkaline phosphate superfamily fold adopted by all S1 sulfatases. The secondary structures α-helix, loop, and β-strand are coloured in red, green, and yellow, respectively. The C-terminal subdomain is coloured cyan, pink, and magenta corresponding to α-helix, loop, and β-strand secondary structures, respectively. Red and black ovals highlight the location of active site and C-terminal subdomain, respectively. (**C**) The proposed transesterification-elimination mechanism for sulfate ester hydrolysis by S1 sulfatases. The initial step involves donation of a proton from the γ1 hydroxyl of the formylglycine (FGly) to an Asp residue, allowing γ1 to attack the incoming sulfate and form a covalent intermediate. Simultaneously, a His residue protonates the oxygen of the scissile linkage meaning a fully protonated carbohydrate exits the active site. Next, abstraction of a proton from γ2 by a His residue generates an aldehyde and results in elimination of the sulfate group. A water molecule then enters the active site and hydrates the FGly aldehyde to a gem diol resetting the catalytic apparatus to the resting state.

The modification of complex glycans through sulfation is a feature of all metazoans [1,2]; the most common of which are colonic mucin *O*-glycans (cMOs) [3] and glycosaminoglycans (GAGs). Mucin, a glycoprotein consisting of *ca*. 80% *O*-glycans by mass, is the major component of the mucus layer that lines all epithelial surfaces, provides protection from the environment, and has roles in cellular regeneration, differentiation, adhesion, and signalling [4]. All mucins possess large protein domains enriched in amino acid repeats of Pro, Ser and Thr (PTS domains) with *O*-glycan attachment occurring on Ser/Thr. The type of mucin changes along the human digestive tract with MUC2 being the dominant form expressed in small intestine and colon [5]. MUC2 is part of the secreted gel-forming group of mucins, along with MUC5AC, MUC5B, and MUC6, and is constitutively expressed by the mucin-producing goblet cells found in the intestinal epithelium [6]. Additionally, the *O*-glycosylation varies along the colon with a marked increase in sulfation from the small intestine to the distal colon where MUC2 is heavily *O*-sulfated – up to 10% by mass [7] (Figure 1A) [5,8,9]. The abundance of sulfated *O*-glycans accompanies an increased bacterial load. Indeed, the human gut microbiota (HGM) in the distal colon contains ∼10^9–10^ cfu/ml, the highest in the body [10]. These bacteria have coevolved with their host to become essential for health, to provide protection from pathogens, and to train and regulate the immune system [11]. The HGM also generates up to 10% of the host’s calories through complex carbohydrate fermentation [12]. In the colon, the sulfated mucin provides both a protective barrier from the HGM, maintaining a healthy distance from the epithelial layer and serves as a colonisation factor and nutrient source for specific microbiota members [13,14]. The HGM has evolved specially adapted carbohydrate sulfatases to degrade and utilise sulfated *O*-glycans from colonic mucins [15].

GAGs occur in the glycocalyx and extracellular matrix (ECM) of almost all animal cells. These glycans interact with both small molecules (such as hormones) and numerous extracellular proteins and regulate extracellular cell signalling, development, and homeostasis [16,17]. The most abundant GAGs are heparan sulfate (HS), heparin (Hep), chondroitin sulfate (CS), and dermatan sulfate (DS) (Figure 1A). HS, the most complex GAG, is a major driver of cell function that interacts with fibroblast growth factors and its sulfation level modulates homeostasis. Indeed, erroneous signalling associated with cancer has been linked to HS-altered sulfatase expression [18]. Hep is expressed in the mast cells of the immune system and is released at sites of injury and inflammation [19]. Hep is used widely as an anticoagulant, although this is not thought to be its biological role [19,20]. CS is present in the ECM where it has been implicated in regulating central nervous system functions [21] and is enriched in joint tissue where it acts as a shock absorber and is essential for joint function [22]. DS is most commonly found in skin and it has been attributed roles as diverse as wound repair, involvement in regulation of blood coagulation, and in the immune response [23].

*Bacteroides* species belong to the Bacteroidetes, a dominant phylum of the HGM and major complex glycan degraders. Species such as *Bacteroides thetaiotaomicron* (*B. theta*) dedicate 15–20% of their genome to carbohydrate metabolism [24]. Members of the Bacteroidetes phyla arrange their carbohydrate active enzymes (CAZymes), including sulfatases, into discrete polysaccharide utilisation loci (PULs) [25]. These systems are sets of colocalised genes that are coregulated in response to particular glycans, which allow the glycan target of a PUL to be predicted based on the relatedness of its CAZymes to characterised examples. Several *Bacteroides* species can degrade and utilise sulfated host glycans [15,26–28]. GAGs sloughed from the colonic epithelial layer, serve as high priority nutrient sources for *B. theta*. Indeed, this substrate is utilised ahead of a number of other carbohydrate sources, such as ingested plant glycans, and their metabolism is not suppressed by glucose [29]. Carbohydrate sulfatases are also known to be essential for the utilisation of GAGs and cMOs by *Bacteroides* [15,26,27]. Additionally, GAG metabolism generates higher levels of short-chain fatty acids acetate and propionate, as well as the neurotransmitter γ-amino butyrate (GABA) [30]. Indeed, high *Bacteroides* populations correlate with depression that is associated with increased GABA levels in the brain [31].

Despite the importance of carbohydrate sulfatases in the metabolism of host glycans by HGM *Bacteroides*, the mechanisms behind their carbohydrate substrate recognition remain relatively understudied. The last 5 years have witnessed intense study of the mechanisms of glycan recognition by sulfatases, in line with their importance to host glycan metabolism and potential roles in diseases such as colorectal cancer [32] and ulcerative colitis (UC) [33], a type of inflammatory bowel disease. Only the carbohydrate sulfatases of *B. theta* [15,26–28] and *Pedobacter heparinus* (*P. heparinus*) [34–36] have been extensively characterised, most work having being carried out on *B. theta*. The present review aims to collate the roles played by bacterial carbohydrate sulfatases in host glycan utilisation and to assess the drivers behind their specificity.

## Classification, active site interactions, and mechanisms of S1 sulfatases

In the SulfAtlas database [37], sulfatases are divided into four families (S1–S4) based on sequence homology. Each family has a conserved fold and catalytic mechanism, but S2–S4 are unique to bacteria [37,38]. Only the S1 family, found in all domains of life, contains carbohydrate sulfatases and is further subdivided by sequence homology into 110 subfamilies (denoted S1_X). The S1 family belongs to the alkaline phosphatase superfamily, possessing an N-terminal α-/-β-/-α fold with a small C-terminal subdomain (Figure 1B). These enzymes utilise a nongenome-encoded FGly as a catalytic nucleophile, which is generated cotranslatory from a conserved Ser/Cys residue, through the action of a FGly-generating enzyme (aerobes) [39,40] or an anaerobic sulfatase-maturing enzyme (anaerobes) [41], in the consensus sequence **C/S-X-P/A-S/X-R** [37,42,43]. A conserved Lys or His acts as the catalytic acid to protonate the leaving group sugar. The sulfate-binding site is invariant, containing an essential calcium ion and the catalytic mechanism is believed to follow a transesterification-elimination pathway [38] (Figure 1C). Briefly, the gem diol form of the FGly residue attacks the sulfate group, after the transfer of a proton from γ1 to a conserved Asp residue, forming a sulfate-enzyme covalent intermediate. Simultaneously, the leaving group carbohydrate is protonated by the catalytic acid and a proton is abstracted from the γ2 hydroxyl-causing aldehyde formation and elimination of the sulfate-enzyme covalent intermediate (Figure 1C). The FGly aldehyde is then hydrated to reform the gem diol and regenerate the catalytic apparatus. The conserved sulfate site is denoted the S subsite and the sugar to which the sulfate group is attached occupies the 0 subsite. As the sugar chain moves towards the reducing end, the subsites increase progressively (+1, +2, +3, etc.), while as the sugar chain moves towards the nonreducing end, subsite numbering correspondingly decreases (–1, –2, –3, etc.). Although the S subsite is invariant across the S1 family, there is considerable variability in the carbohydrate-binding regions that drive the observed exquisite specificity.

To date, a significant number of bacterial carbohydrate sulfatases identified have been shown to be *exo*-acting [15,26,34,35,44]. The active site of these sulfatases is located in a pocket that only recognises sulfate groups from the nonreducing end of sulfated glycans. *Exo*-acting sulfatases are unable to access sulfate groups that are located internally within the glycan chains and require other CAZymes to make these sulfate groups accessible. By contrast, *endo*-acting carbohydrate sulfatases can remove sulfate groups that are internally located with the glycan chain and may not need other CAZymes to process the substrate [15,27].

## S1 carbohydrate sulfatases involved in cMOs desulfation

**Figure 2:**
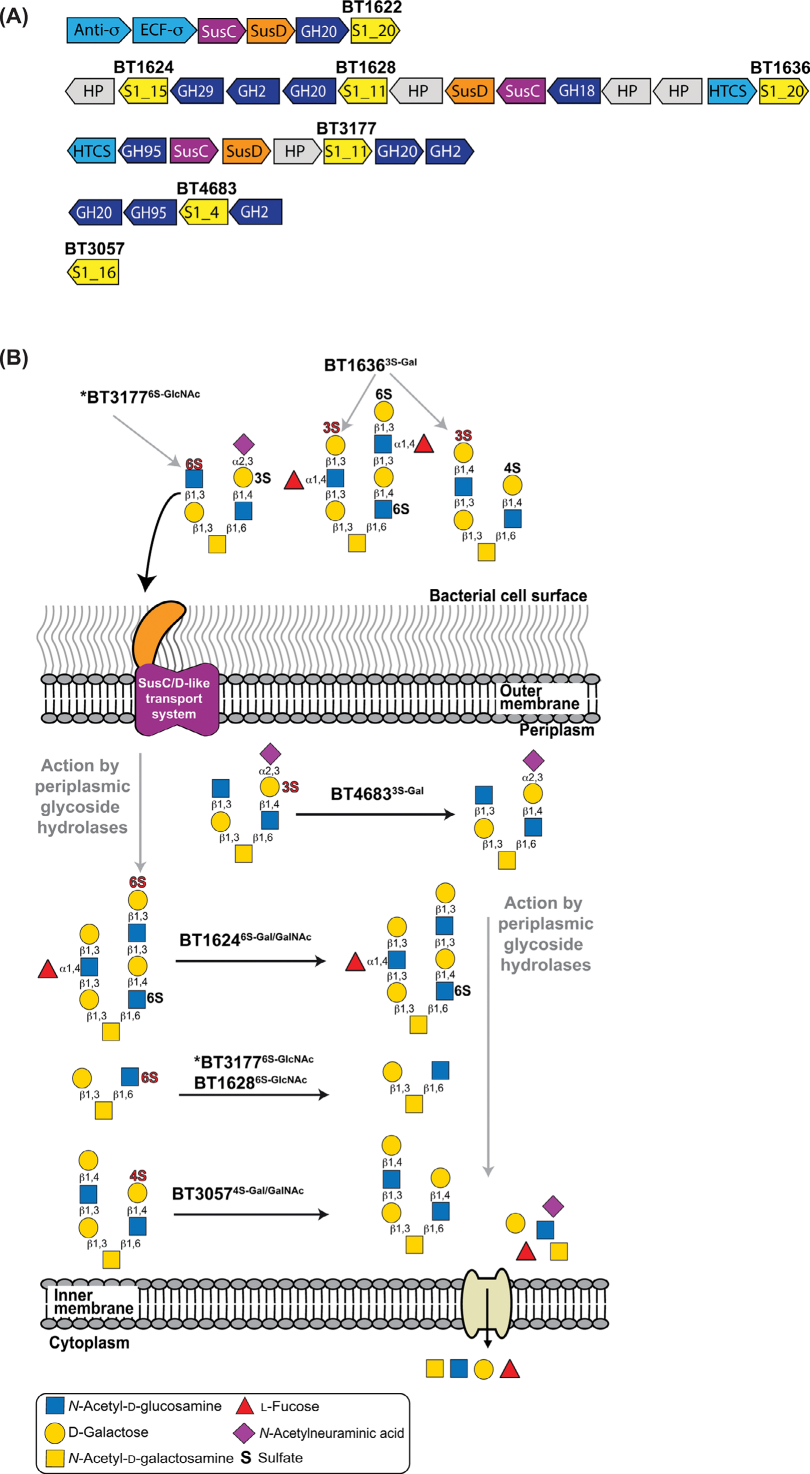
cMOs PULs in *B. theta* and the proposed point of action of the encoded sulfatases (**A**) cMOs PUL structure in *B. theta*. The PUL components are as follows: HP (hypothetical protein), S1 (sulfatase S1 with the respective subfamily number), GHXX (glycoside hydrolase with X representing the family number), HTCS (hybrid two-component system), SusC (starch utilisation system C-like), SusD (starch utilisation system D-like), ECF-σ (extracytoplasmic factor sigma), and anti-σ (antisigma factor). (**B**) Shows a model of the *B. theta* cell and identifies the degradative step at which the currently characterised *B. theta* carbohydrate sulfatases have been proposed to act. The glycans shown are models containing all appropriate linkages based on the identified activities of the sulfatases. Only the cellular location of BT1636^3S-Gal^ has been experimentally proven and the location of the remaining carbohydrate sulfatases is based on the signal peptide sequence and predictions by SignalP 5.0. An asterisk (*) indicates that, due to BT3177^6S-GlcNAc^ containing an SPII signal peptide, it is predicted to be membrane bound; however, it is not known whether this is within the periplasmic space or at the extracellular surface.

Microbiota sulfatase activity has been shown to be correlated with disease states in animal models and humans. The model HGM organism *B. theta* drives sulfatase-dependent colitis in a susceptible mouse model [33]. In humans, the increased carbohydrate HGM sulfatase activity correlates with UC severity [45] and patients with active UC have decreased mucin sulfation [46,47]. Sulfatases have therefore come under scrutiny as potential targets to treat UC. Recently, several *B. theta* sulfatases implicated in colonic mucin degradation have been biochemically and structurally characterised [15,28]. *B. theta* uses sulfatases from at least five S1 subfamilies, S1_4, S1_11, S1_15, S1_16, and S1_20 to desulfate all of the known sulfoester linkages in mucin [15]. These sulfatases are distributed across at least four PULs (Figure 2A) and can act throughout the *O-*glycan degradative process (Figure 2B). Henceforth, carbohydrate sulfatases will be identified by their locus tag and activity in superscript.

Although *B. theta* encodes multiple *O*-glycans active sulfatases, a single enzyme has emerged as critical to its ability to utilise cMOs. The key S1_20 sulfatase, BT1636^3S-Gal^, is located at the bacterial surface [15]. The removal of *O*3 sulfation on terminal d-Galactose (Gal) residues by BT1636^3S-Gal^ is required to initiate the degradation of cMOs at the cell surface (Figure 2B). The S1_4 enzyme BT4683^3S-Gal^ is an *endo*-acting sulfatase that removes internal *O*3 sulfates from Gal on intact cMOs [15]. Whilst the S1_11 enzymes, BT1628^6S-GlcNAc^ and BT3177^6S-GlcNAc^, are *exo*-acting from the nonreducing end on intact cMOs, removing *O*6 sulfation from *N*-acetyl-D-glucosamine (GlcNAc). These enzymes cannot access internal 6S-GlcNAc residues and require other CAZymes to process the cMOs further [15]. Indeed, none of the remaining characterised sulfatases were active against intact cMOs and only activity against sulfated monosaccharides has been demonstrated [15] (Figure 2B). These *exo*-acting enzymes may work on terminally sulfated di-, tri- or tetrasaccharides generated by the action of mucinolytic glycoside hydrolases (GH). The S1_15 enzyme BT1624^6S-Gal/GalNAc^ demonstrated comparable activity and binding towards both *O*6-sulfated Gal and *N*-acetyl-d-galactosamine (GalNAc) [15,28]. The S1_16 enzyme BT3057^4S-Gal/GalNAc^ showed a similar activity and binding towards both 4S-Gal and 4S-GalNAc [28]. It should be noted that *O*4 sulfation has not been formally observed in cMOs and it is likely that BT3057^4S-Gal/GalNAc^ is utilised to desulfate additional mucin *O*-glycans that arrive in the colon such as those of the saliva. Finally, BT1622^3S-Gal/GalNAc^ preferentially desulfates 3S-GalNAc. Interestingly, although it is known that mucin can be sulfated in Gal, there are no reports of this substitution being linked to GalNAc [15].

### Desulfation of 3-O-sulfated galactose and *N*-acetyl-d-galactosamine

**Figure 3:**
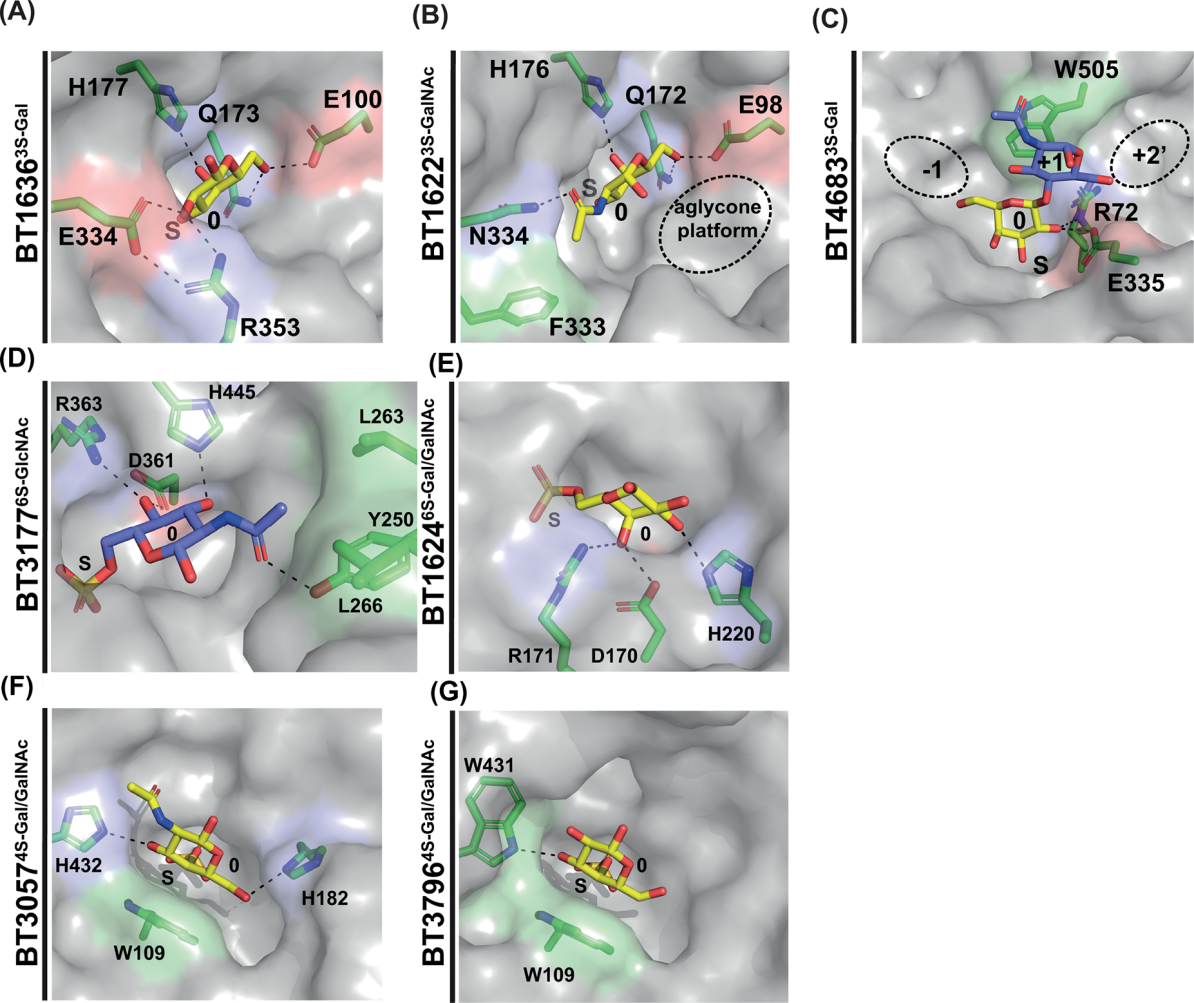
Key carbohydrate recognition features of cMOs sulfatases (**A**) The 0 subsite interactions of the S1_20 enzyme BT1636^3S-Gal^. (**B**) The 0 subsite interactions of the S1_20 enzyme BT1622^3S-Gal/GalNAc^. (**C**) The 0 and +1 subsite interactions of the *endo*-acting S1_4 enzyme BT4683^3S-Gal^, additional potential +1 and +2’ subsites are indicated in black ovals. (**D**) The 0 subsite interactions of the S1_11 enzyme BT3177^6S-GlcNAc^. (**E**) The 0 subsite interactions of the S1_15 enzyme BT1624^6S-GalNAc^. (**F**,**G**) The 0 subsite interactions of the S1_16 enzymes BT3057^4S-Gal/GalNAc^ and BT3796^4S-Gal/GalNAc^. Aromatic and hydrophobic residues are highlighted in pale green, basic, and amine residues in light blue, and acidic residues in pale red. S indicates the invariant sulfate catalytic site and 0 indicates the carbohydrate-binding subsite to which the scissile sulfate is attached.

BT1636^3S-Gal^ is an essential enzyme for *B. theta* to efficiently utilise cMOs. This *exo*-acting enzyme utilises a His residue to recognise the axial *O*4 of Gal, the epimeric position that distinguishes D-galactose from D-glucose (Glc), a feature that is well conserved across the S1_20 subfamily [15] (Figure 3A). High affinity for Gal is further driven by strong interactions with *O*2 through Glu and Arg amino acid residues, a feature found in ∼2/3 of subfamily members. BT1636^3S-Gal^ makes no obvious interactions beyond the 0 subsite, but the addition of fucose to sulfated Lewis antigen motifs significantly lowers the activity of the enzyme [15].

The second S1_20 member expressed by *B. theta* is *exo*-acting BT1622^3S-Gal/GalNAc^ that contains similar 0 subsite interactions to BT1636^3S-Gal^ but, lacks the Glu and Arg amino acid residues that interact with *O*2 (Figure 3B). Instead, BT1622^3S-Gal/GalNAc^ has a more open pocket enabling it to accommodate the sugar 3S-GalNAc, with its bulkier C2 *N*-acetyl compared with the hydroxyl of Gal [15] (Figure 3B). Indeed, BT1622^3S-Gal/GalNAc^ is tenfold more active against 3S-GalNAc than 3S-Gal-containing substrates. The role of BT1622^3S-Gal/GalNAc^ is not clear, since its biological substrate has not been identified. Structural studies, including the product α-GalNAc, suggest that it could target an *O*3-sulfated Tn antigen. There is a more open landscape into which the α anomeric hydroxyl points that could potentially accommodate an amino acid chain. Such sulfation of the Tn antigen would restrict mucin core production to sulfated 6 or 7 type. However, these sulfated structures remain to be identified in biological samples.

The 0 subsite of BT4683^3S-Gal^ only interacts with the *O*2 of Gal via Glu and Arg [15]. The Glu residue is orthologous to that in the S1_20 enzymes, unlike the Arg residue which occupies a similar location, but originates from the N-terminus. BT4683^3S-Gal^ also has aromatic stacking at +1 to facilitate GlcNAc binding and has little affinity for Gal monosaccharides [15] (Figure 3C). The +1 aromatic-stacking interaction is a rare adaptation largely absent from homologues, so it is likely that these utilise multiple weak-binding sites to achieve substrate binding [15]. This would be consistent with the *endo*-activity of BT4683^3S-Gal^, which has an open cleft to accommodate multiple sugars.

### Desulfation of 6-O-sulfated GlcNAc

The structure of BT3177^6S-GlcNAc^ complexed with 6S-GlcNAc revealed a recognition triad. A His co-ordinates *O*3 and a Asp/Arg interact with *O*4 (Figure 3D). These residues are highly conserved across the S1_11 subfamily [28] (91% and 98%, respectively); however, a lack of conservation is observed in the residues that recognise the *C*2 *N*-acetyl group. This region is absent from around half of the subfamily, especially those from aquatic environments that employ the same triad to recognise L-galactose [28,48]. In BT3177^6S-GlcNAc^, however, this region contains two Leu and a Tyr residue, providing a hydrophobic environment to interact with the methyl group of the acetyl moiety (Figure 3D). In comparison, the paralog BT4656^6SGlcNAc/GlcNS^, which operates in GAG metabolism, is highly charged in this region [28] (discussed later), demonstrating its high evolutionary plasticity.

### Desulfation of 6-O-sulfated *N*-acetyl-d-galactosamine

Four *B. theta* S1_15 sulfatases that desulfate 6S-*galacto*-configured substrates, such as 6S-Gal and 6S-GalNAc, have been described: BT1624^6S-Gal/GalNAc^, BT3109^6S-Gal^, BT3333^6S-GalNAc^, and BT4631^6SGal/GalNAc^. The presence of BT1624^6S-Gal/GalNAc^ in a mucin-associated PUL containing BT1628^6S-GlcNAc^ and BT1636^3S-Gal^ link it to cMO metabolism (Figure 2A). Similar to S1_11, S1_15 subfamily members utilise a recognition triad. A His co-ordinates *O*3 and an Asp/Arg dyad co-ordinates *O*4 (Figure 3E). In S1_15, however, these residues originate from the N-terminus and the Gal/GalNAc substrate sits in the 0 subsite perpendicular relative to GlcNAc in S1_11 [28]. Thus, despite seemingly similar recognition triads, the interactions are spatially unique and specificity for D-*galacto*- versus D-*gluco*-configured substrates is absolute. It remains formally possible that S1_15 may be able to recognise L-glucose in the same way that S1_11 recognises both D-GlcNAc and L-galactose but, to the best of our knowledge, this remains untested.

### Desulfation of 4-O-sulfated *N*-acetyl galactosamine

Two *B. theta* S1_16 sulfatases, BT3057^4S-Gal/GalNAc^ and BT3796^4S-Gal/GalNAc^, can desulfate both 4S-Gal and 4S-GalNAc monosaccharides with equal efficiency [15]. Both employ a critical Trp at the 0 subsite to stack against the α face of Gal and a secondary amine to co-ordinate *O*3 [28] (Figure 3F,G). Loss of the Trp residue interaction, through mutation to Ala, abolishes activity. Although this residue is only conserved in 37% of S1_16 sequences, it is present in 84% of sequences from the HGM, suggesting that most HGM S1_16 enzymes are 4S-Gal/GalNAc sulfatases [28].

BT3796^4S-Gal/GalNAc^ is not in a PUL associated with mucin metabolism and BT3057^4S-Gal/GalNAc^ is an orphan gene. It is common for PULs to utilise orphan genes to augment existing capacity, as in both GAG PULs [26,27] (described below) and we have speculated that BT3057^4S-Gal/GalNAc^ is the mucin *O*-glycan 4S-Gal sulfatase (Figure 2B). A role for BT3796^4S-Gal/GalNAc^, or the possibility that neither enzyme participates, cannot be excluded.

### Summary

S1 carbohydrate sulfatases are essential for *B. theta* to utilise cMOs and act at all stages of cMO metabolism, initiating transport into the periplasm, acting on intermediate structures, and desulfating final monosaccharide products. Each S1 sulfatase subfamily has a unique, nonredundant role, indicating that the degradation of cMOs by *B. theta*, and potentially other mucin-utilising *Bacteroides* species, could be interrupted. This makes carbohydrate sulfatase-degrading cMOs attractive drug targets, especially those that are *exo*-acting with a tight, and specific, substrate-binding pocket.

## Desulfation of GAGs

### Hep and HS

HS is a linear polysaccharide composed of, 1,4-linked, repeating disaccharides comprising a uronic acid (either β-D-glucuronic acid [GlcA] or α-L-iduronic acid [IdoA]) and α-D-glucosamine (D-GlcN) (Figure 1A). The uronate can be 2-*O*-sulfated and the glucosamine can be *N*-acetylated (GlcNAc), *N*-sulfated and *O*-sulfated at position 6 and, less commonly, at position 3 [1]. The variable composition and sulfation provide several thousand sequence permutations for a short oligosaccharide, making HS one of the most complex glycans known. HS has regions that are heavily sulfated (S domains), regions of low- (NA domains) and intermediate sulfation domains (NS domains) [1,17]. It shares the same underlying composition and linkage geometry with Hep but, the latter is more sulfated, has a higher proportion of IdoA and tends to be much smaller in size (5–10 kDa compared with up to 100 kDa of HS).

**Figure 4:**
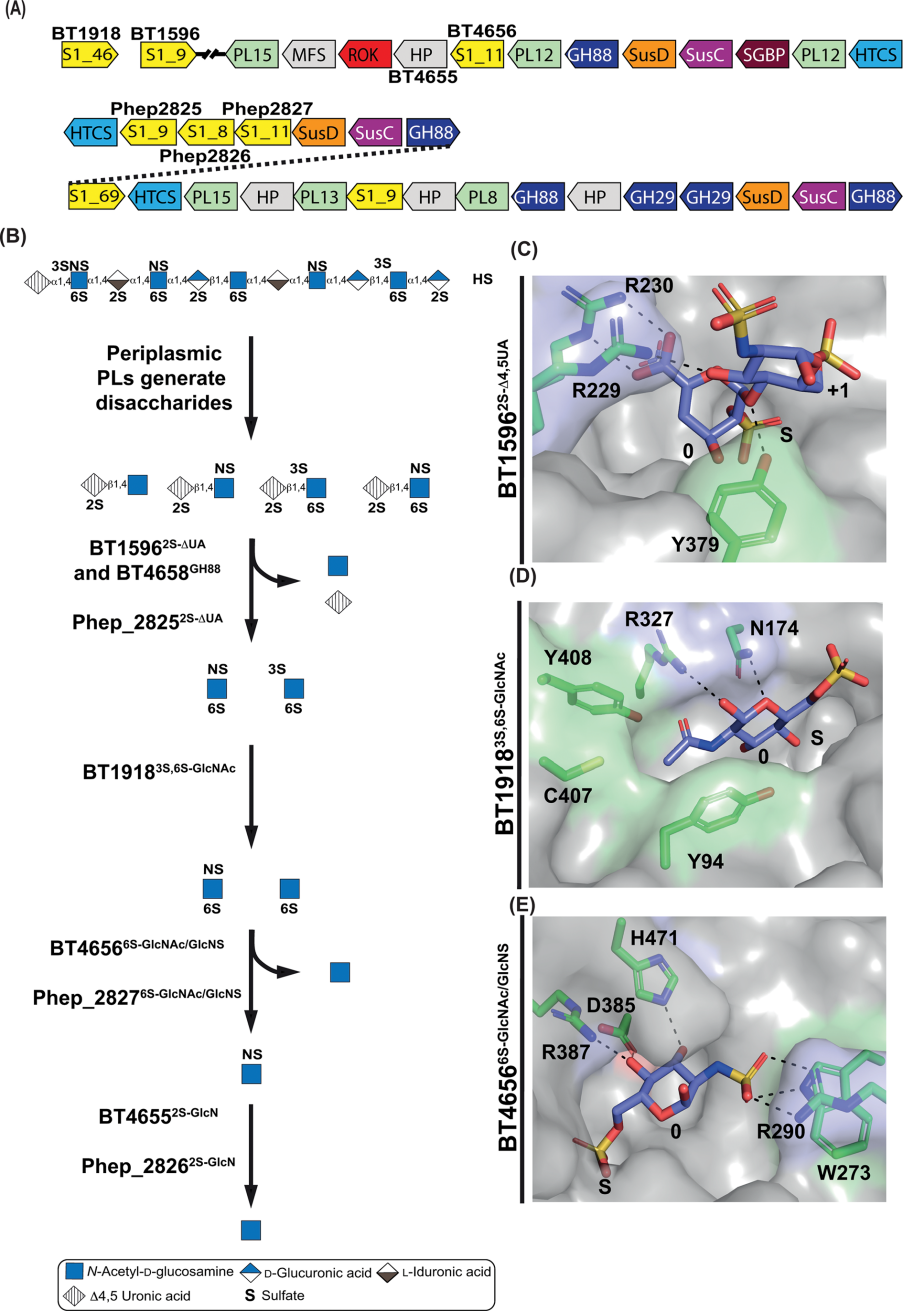
Genomic context, functional and structural details of Hep/HS-degrading sulfatases (**A**) The Hep/HS PULs of *B. theta* and *P. heparinus*. HP (hypothetical protein), S1 (sulfatase S1 with the respective subfamily number), GHXX (glycoside hydrolase with X representing the family number), PLXX (polysaccharide lyase with X representing the family number), DUF (domain of unknown function), HTCS (hybrid two-component system), SusC (starch utilisation system C-like), SusD (starch utilisation system D-like), SGBP (surface glycan-binding protein), MFS (major facilitator superfamily), and ROK (repressor, ORF, kinase superfamily). (**B**) The role of carbohydrate sulfatases in the Hep/HS degradative pathway. (**C**) The 0 and +1 subsite carbohydrate recognition features of the S1_9 enzyme BT1596^2S-Δ4,5UA^. (**D**) The 0 subsite carbohydrate recognition features of the S1_46 enzyme BT1918^3S,6S-GlcNAc^. (**E**) The 0 subsite carbohydrate recognition features of the S1_11 enzyme BT4656^6S-GlcNAc/GlcNS^. Aromatic and hydrophobic residues are highlighted in pale green, basic and amine residues in light blue, and acidic residues in pale red. S indicates the invariant sulfate catalytic site and 0 indicates the carbohydrate-binding subsite to which the scissile sulfate is attached.

Two Bacteroidetes species, *P. heparinus* (formerly *Flavobacterium heparinum*) from soil [34–36] and *B. theta* [26,44] have had their HS/Hep PULs characterised (Figure 4A). Their S1 sulfatases are periplasmic, acting sequentially in the final stages of HS/Hep metabolism. *P. heparinus* uses three exo-acting S1 sulfatases from S1_8, S1_9, and S1_11. The S1_9 enzyme, Phep_2825^2S-Δ4,5UA^, first acts on disaccharides produced by polysaccharide lyases (PL), to remove *O*2 sulfation [36], allowing the glycoside hydrolase Phep_2830^GH88^ from glycoside hydrolase family 88 (GH88), to cleave the disaccharide into monosaccharides; an essential step that enables the S1_11 enzyme, Phep_2827^6S-GlcNAc^, to desulfate 6S-GlcNS to GlcNS [34]. The S1_8 enzyme Phep_2826^2S-GlcN^, which is inhibited by *O*6 sulfation, then acts on GlcNS to produce GlcN [35] (Figure 4B). No *O*3 sulfatase has been identified in *P. heparinus* able to remove *O*3 sulfation from 3S,6S-GlcNAc or 3S-GlcNS.

In *B. theta*, three exo-acting periplasmic S1 sulfatases from subfamilies S1_9, S1_11, and S1_46 participate in HS/Hep catabolism. The S1_9 enzyme BT1596^2S-Δ4,5UA^ acts in an analogous manner to Phep_2825^2S-Δ4,5UA^ [44]. Sulfate at *O*3 on 3S,6S-GlcNAc is removed by the S1_46 sulfatase, BT1918^3S,6S-GlcNS/GlcNAc^, generating 6S-GlcNAc [28], which is then desulfated by the S1_11 enzyme BT4656^6S-GlcNS/GlcNAc^. BT4656^6S-GlcNS/GlcNAc^ is capable of desulfating both 6S-GlcNAc and 6S-GlcNS but cannot tolerate *O*3 sulfation [26,44]. *D*e-*N*-sulfation of GlcNS is performed by BT4655, but its point of action in the pathway is unknown (Figure 4B). BT4655 has not been biochemically characterised, but a gene deletion mutant of BT4655 (Δ*bt4655*) was grown on Hep, HS, and desulfated-Hep, showing a ∼50%, ∼25%, and no reduction in growth, respectively, correlating broadly with the proportion of GlcNS in each polysaccharide [26]. Analysis of Δ*bt4655* spent growth media on Hep showed that GlcNS remained in the media, whereas it was absent from wild-type spent growth media [26]. These data support the role of BT4655 as a novel sulfatase family. Since this enzyme family has not been biochemically characterised, it is not part of the SulfAtlas database.

All three S1 HS/Hep sulfatases from *B. theta* have been structurally characterised and their specificity drivers elucidated. BT1596^2S-ΔUA^ desulfates at position-2 of all disaccharides generated by the PLs of PUL_Hep/HS_, acting on IdoA and GlcA to produce the same product, a 4,5-unsaturated uronic acid (Δ4,5UA). The 0 subsite of BT1596^2S-Δ4,5UA^ is specific for Δ4,5UA and employs the carboxylate of the 0 subsite uronate as a major specificity determinant, forming bidentate interactions via Arg230 (Figure 4C). Beyond the 0 subsite, Tyr379 interacts with the glycosidic bond and Arg229 co-ordinates *O*3, affecting substrate affinity, but not catalytic activity (Figure 4C). BT1596^2S-Δ4,5UA^ precedes the GH88, BT4658^GH88^, allowing it to generate sulfated monosaccharide substrates for subsequent sulfatases [26].

BT1918^3S,6S-GlcNAc^ desulfates 3S,6S-GlcNAc at position-3 requires the *N*-acetyl group for activity and cannot desulfate 3S,6S-GlcN. The *N*-acetyl group sits in a hydrophobic pocket formed by the phenol rings of two Tyr residues and methylene backbone of Arg (Figure 4D); further interactions being provided by the endocyclic ring oxygen to an Asn [28]. These interactions are invariant in *Bacteroides* species of the HGM. BT1918^3S,6S-GlcNAc^ cannot tolerate *N*-sulfation in place of *N*-acetylation, implying that *B. theta* may encounter relatively little 3S,6S-GlcNS compared with 3S,6S-GlcNAc, bearing in mind that 3S sulfation is itself a rare modification [49].

The de-6-O-sulfating sulfatase BT4656^6S-GlcNAc/GlcNS^ utilises the same recognition triad as BT3177^6S-GlcNAc^ to bind GlcNAc (Figure 4E), but the area that co-ordinates *C*2 substituents is much more positively charged [28]. Unlike cMOs, HS/Hep contain 6S-GlcNS and to accommodate this, BT4656^6S-GlcNAc/GlcNS^ has replaced the hydrophobic region in BT3177^6S-GlcNAc^ with an Arg that stacks on a Trp through cation–π interactions (Figure 4E). The Arg forms a bidentate interaction with the *N*-linked sulfate, while the indole nitrogen of Trp co-ordinates the third oxygen of the sulfate; these features are conserved in other BT4656 homologues present in HS/Hep PULs [28]. The fact BT4656^6S-GlcNAc/GlcNS^ has special adaptations for 6S-GlcNS suggests that BT4655 acts after BT4656^6S-GlcNAc/GlcNS^.

### Summary

In contrast with cMO catabolism, all sulfatases in HS/Hep degradation are *exo*-acting and strictly hierarchal. Additional capacity is provided to PUL_Hep/HS_ by orphan genes BT1596^2S-ΔUA^ (shared with PUL for CS/DS metabolism, see below) and BT1918^3S,6S-GlcNAc^. The S1_11 enzyme BT4656^6S-GlcNAc/GlcNS^ has specialist adaptations for HS/Hep sulfation, not observed in the cMO S1_11 BT3177^6S-GlcNAc^, but conserved in HGM orthologues in HS/Hep PULs. *B. theta*, in contrast with *P. heparinus*, which uses an S1_8 enzyme to desulfate GlcNS, utilises a novel sulfatase family that is, again, conserved in homologous HGM Bacteroidetes HS/Hep PULs.

### Chondroitin and DS

CS and DS are linear polysaccharides composed of repeating β-1,4-linked disaccharides of a uronic acid (β-D-GlcA in CS and α-L-idoA acid in DS) and 1,3 linked to β-GalNAc. In CS, sulfation can occur on *O*2 of GlcA and at positions 4 or 6 of GalNAc. In DS, sulfation is exclusive to *O*4 of GalNAc (Figure 1A).

**Figure 5:**
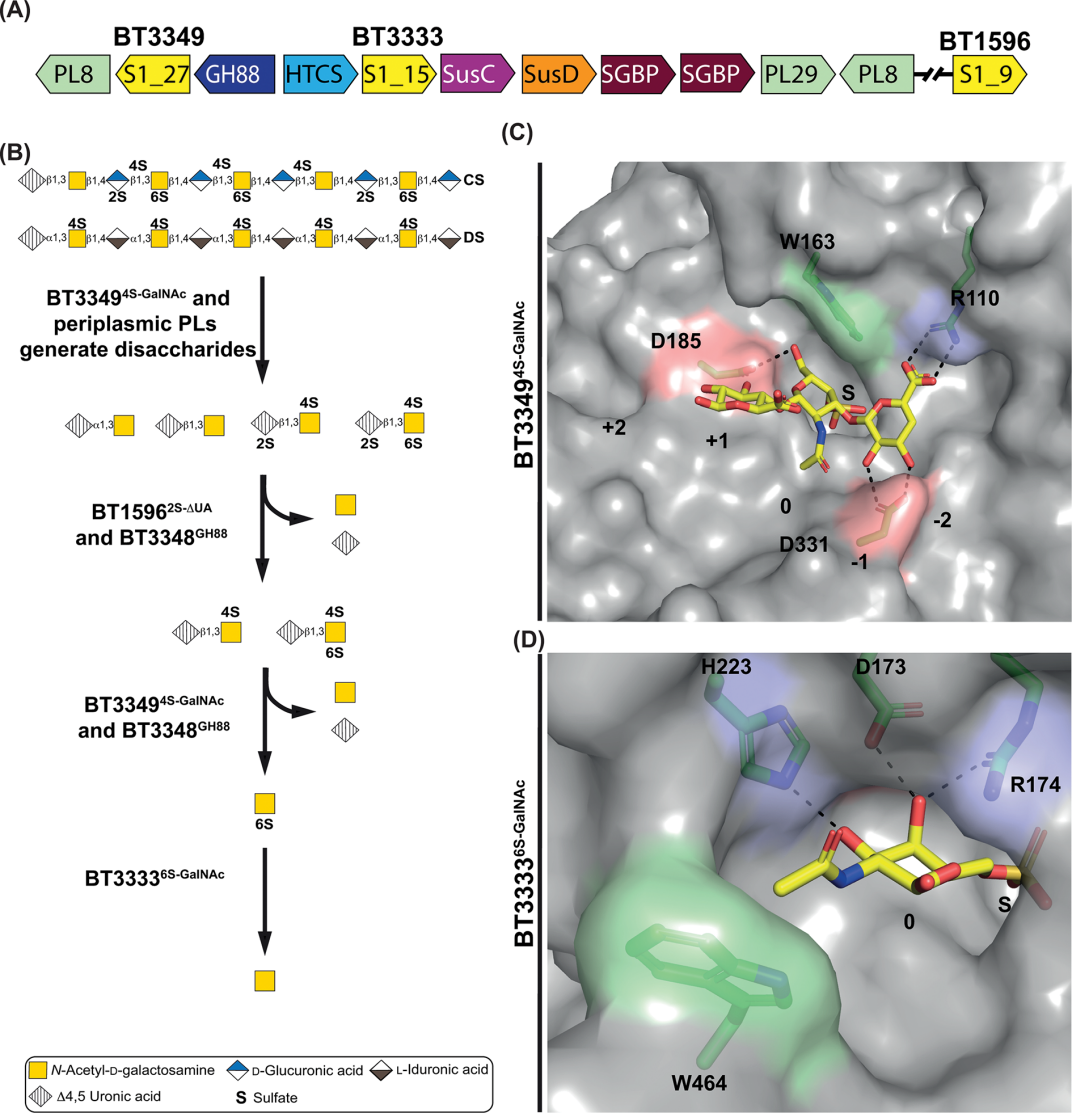
Genomic context, functional, and structural details of chondroitin and DS-degrading sulfatases (**A**) The chondroitin/DS PULs of *B. theta*. The PUL components are as follows: S1 (sulfatase S1 with the respective subfamily number), GHXX (glycoside hydrolase with X representing the family number), PLXX (polysaccharide lyase with X representing the family number), HTCS (hybrid two-component system), SusC (starch utilisation system C-like), SusD (starch utilisation system D-like), SGBP (surface glycan-binding protein). (**B**) The role of S1 carbohydrate sulfatases in the chondroitin/DS degradative pathway. (**C**) The –1, 0, and +1 subsite carbohydrate recognition features of the S1_27 enzyme BT3349^4S-GalNAc^. (**D**) The 0 subsite carbohydrate recognition features of the S1_11 enzyme BT3333^6S-GalNAc^. Aromatic and hydrophobic residues are highlighted in pale green, basic and amine residues in light blue, and acidic residues in pale red. S indicates the invariant sulfate catalytic site and 0 indicates the carbohydrate-binding subsite to which the scissile sulfate is attached.

The PUL responsible for CS and DS metabolism, as well as the accessory proteins, has been characterised in *B. theta* [26] (Figure 5A). Three S1 sulfatases, BT3349^4S-GalNAc^, BT3333^6S-GalNAc^, and BT1596^2S-Δ4,5UA^ from subfamilies S1_27, S1_15, and S1_9, respectively, perform CS and DS desulfation in *B. theta*. BT1596^2S-ΔUA^, described above, can de-2-*O*-sulfate disaccharides produced from both CS/DS and HS/Hep [26,27]. All three sulfatases are periplasmic but, while BT1596^2S-Δ4,5UA^ and BT3333^6S-GalNAc^ are *exo*-acting, BT3349^4S-GalNAc^ is *endo*-acting and contains an SpII signal peptide, suggesting membrane association [27,44]. BT3349^4S-GalNAc^ acts on intact CS and DS prior to PL action but, its activity is significantly reduced by *O*2 sulfation and also, to a lesser degree, *O*6 sulfation [44] (Figure 5B). BT3349^4S-GalNAc^ may therefore act on regions of CS and DS containing only *O*4 sulfation and act simultaneously with, or prior to, PLs. CS that contains *O*2, *O*4, and *O*6 sulfation, may hinder BT3349^4S-GalNAc^ activity to the extent that the PLs act first producing disaccharides for BT1596^2S-Δ4,5UA^ to de-2-*O*-sulfate, thereby allowing BT3349^4S-GalNAc^ to operate more efficiently. Only after tri- and disulfated disaccharides have had both *O*2 and *O*4 sulfation removed by BT1596^2S-Δ4,5UA^ and BT3349^4S-GalNAc^, respectively, is the GH88 enzyme BT3448^GH88^ able to produce the monosaccharide product 6S-GalNAc. BT3333^6S-GalNAc^, then desulfates 6S-GalNAc to produce GalNAc [27] (Figure 5B).

The structure of BT3349^4S-GalNAc^ reveals that the glycan chain runs across the surface of the protein enabling its *endo* activity, and exhibits two major glycan-binding sites, a 0 subsite and a –1 subsite. The former utilises a Trp for aromatic stacking and an Asp to co-ordinate *O*6 (Figure 5C), while the latter is an ionic pincer, an Asp co-ordinating *O*2 and *O*3, and Arg forming a bidentate interaction with the carboxylate, locking the GlcA residue in place (Figure 5C). The strong interaction of Asp331 with *O*2 of the –1 GlcA explains why *O*2 sulfation causes major reduction in activity as it would cause both steric and charge repulsion (Figure 5C). Additionally, *O*6 sulfation of the 0 subsite GalNAc also causes some loss of activity since it must fit between a hydrophobic Trp and a negatively charged Asp [27] (Figure 5C).

The structure of BT3333^6S-GalNAc^, an enzyme that can desulfate 6S-Gal but has a preference for 6S-GalNAc, reveals a deep pocket where the substrate sits, justifying an *exo*-mode of action. A *galcto-*recognition triad of His, Asp, and Arg co-ordinate *O*3 and *O*4, identical with BT1624^6S-Gal/GalNAc^ and similar to S1_11 enzymes described previously [27] (Figure 5D). The unique driver for GalNAc recognition is a Trp residue co-ordinating the *N*-acetyl group (Figure 5D). Interestingly, this feature is absent from the S1_15 cMO-targeting enzyme BT1624^6S-Gal/GalNAc^, but is conserved in S1_15 sulfatases in CS PULs amongst numerous *Bacteroides* species [28].

### Summary

The sulfatases involved in CS/DS catabolism utilise both *endo*- and *exo*-modes of action. All enzymes are periplasmic, but the rationale for the *endo-*mode of BT3349^4S-GalNAc^ is not entirely clear, nor is its predicted membrane attachment. BT3349^4S-GalNAc^ can desulfate both CS and DS disaccharides and polymers, but its activity is decreased with high levels of *O*2 and *O*6 sulfation, permitting the production of di- and trisulfated disaccharides by periplasmic PLs. BT1596^2S-Δ4,5UA^ will then remove *O*2-sulfates, allowing BT3349^4S-GalNAc^ to act more efficiently. Both *O*2- and *O*4-sulfation must be removed before the GH88 can generate the monosaccharide substrate 6S-GalNAc for BT3333^6S-GalNAc^ for completion of the catabolic process.

## Overall summary

Sulfated host glycans are critical nutrient sources for the *Bacteroides* of the HGM and S1 carbohydrate sulfatases are essential enzymes [15,50]. Most of the S1 carbohydrate sulfatases discussed here are periplasmic in their cellular location. This is in keeping with the PUL degradation model where most glycan degradation is performed in the periplasm, thus maximising energy extraction rather than losing it to the wider community as so called ‘public goods’ [51,52]. This cellular location is also required for the *exo*-acting carbohydrate sulfatases targeting internally located sulfate groups as they require the action of other CAZymes to expose their target substrate. In contrast with the metabolism GAGs, the catabolism of cMOs requires *exo*-acting carbohydrate sulfatases at the bacterial cell surface to process the glycans prior to import into the periplasm [15]. The evolutionary rationale for this is currently not clear, but could be connected with how the SusC/D transport machinery recognises terminal Gal residues in cMOs requiring them to be unsulfated.

S1 family members are CAZymes capable of exquisite substrate recognition involving both sulfate position and sugar stereochemistry. Each S1 subfamily possesses recognition features conserved throughout the subfamily, correlating with the genomic and environmental context of the enzyme [15,28]. Together with their largely *exo*-mode of action, S1 carbohydrate sulfatases involved in cMO metabolism are attractive drug targets that are amenable to small-molecule screening and subsequent engineering for the binding pocket. Inhibition of carbohydrate sulfatases, essential for GAG metabolism by the HGM whose role is not well-understood, will provide new ways to control Bacteroidetes growth, as well as ways to explore the influence of Bacteroidetes in the HGM ecosystem. Owing to their high specificity, carbohydrate sulfatases will also enable examination of complex glycan sulfation in healthy and diseased states.

## Summary

- Bacterial carbohydrate sulfatases targeting host glycans are almost exclusively found in the S1 family of sulfatases.
- S1 carbohydrate sulfatases are exquisitely specific enzymes, demonstrating tailored adaptations to their carbohydrate substrate that can be mapped at a subfamily level.
- The metabolism of HS by soil-dwelling *P. heparinus* and HGM resident *B. theta* largely share common catabolic steps but differ in how some specific sulfoester linkages are targeted.
- The HGM organism *B. theta* can deploy sulfatases to remove every sulfate ester linkage identified in cMOs to date.
- The exquisite specificity of *exo*-acting S1 carbohydrate sulfatases, and their critical importance in cMOs metabolism, makes these enzymes attractive potential drug targets for the treatment of chronic bowel diseases, such as UC.

## Open Access

Open access for this article was enabled by the participation of University of Liverpool in an all-inclusive *Read & Publish* agreement with Portland Press and the Biochemical Society under a transformative agreement with JISC.

## Abbreviations

CAZyme: carbohydrate active enzyme
cMOs: colonic mucin O-glycans
CS: chondroitin sulfate
D-GlcN: α-D-glucosamine
DS: dermatan sulfate
ECM: extracellular matrix
FGly: formylglycine
GABA: γ-amino butyrate
GAG: glycosaminoglycan
Gal: galactose
GH: glycoside hydrolase
GlcNAc: N-acetyl-D-glucosamine
GHXX: glycoside hydrolase with X representing the family number
GH88: glycoside hydrolase family 88
Hep: heparin
HGM: human gut microbiota
HP: protein of unknown function
HS: heparan sulfate
HTCS: hybrid two-component system
IdoA: L-iduronic acid
PL: polysaccharide lyase
PTS: Pro, Ser and Thr
PUL: polysaccharide utilisation loci
S1: sulfatase S1 with the respective subfamily number superscript
SGBP: surface glycan binding protein
SusC: starch utilisation system C-like
SusD: starch utilisation system D-like
UC: ulcerative colitis
